# Epigenome-wide association study of long-term psychosocial stress in older adults

**DOI:** 10.1080/15592294.2024.2323907

**Published:** 2024-03-03

**Authors:** Lauren A. Opsasnick, Wei Zhao, Lauren L. Schmitz, Scott M. Ratliff, Jessica D. Faul, Xiang Zhou, Belinda L. Needham, Jennifer A. Smith

**Affiliations:** a Department of Epidemiology, School of Public Health, University of Michigan, Ann Arbor, MI, USA; b Survey Research Center, Institute for Social Research, University of Michigan, Ann Arbor, MI, USA; c Robert M. La Follette School of Public Affairs, University of Wisconsin-Madison, Madison, WI, USA; d Department of Biostatistics, School of Public Health, University of Michigan, Ann Arbor, MI, USA

**Keywords:** Social epigenomics, psychosocial stress, epigenome-wide association study, DNA methylation, mediation analysis, health behaviours

## Abstract

Long-term psychosocial stress is strongly associated with negative physical and mental health outcomes, as well as adverse health behaviours; however, little is known about the role that stress plays on the epigenome. One proposed mechanism by which stress affects DNA methylation is through health behaviours. We conducted an epigenome-wide association study (EWAS) of cumulative psychosocial stress (*n* = 2,689) from the Health and Retirement Study (mean age = 70.4 years), assessing DNA methylation (Illumina Infinium HumanMethylationEPIC Beadchip) at 789,656 CpG sites. For identified CpG sites, we conducted a formal mediation analysis to examine whether smoking, alcohol use, physical activity, and body mass index (BMI) mediate the relationship between stress and DNA methylation. Nine CpG sites were associated with psychosocial stress (all *p* < 9E–07; FDR q < 0.10). Additionally, health behaviours and/or BMI mediated 9.4% to 21.8% of the relationship between stress and methylation at eight of the nine CpGs. Several of the identified CpGs were in or near genes associated with cardiometabolic traits, psychosocial disorders, inflammation, and smoking. These findings support our hypothesis that psychosocial stress is associated with DNA methylation across the epigenome. Furthermore, specific health behaviours mediate only a modest percentage of this relationship, providing evidence that other mechanisms may link stress and DNA methylation.

## Introduction

Psychosocial stress, or the perception of threat that results in discomfort and emotional tension, can be triggered by a multitude of stressors, including difficulty in relationships, financial uncertainty, or adverse life events such as job loss, discrimination, or health issues [1,2]. Previous research has shown that elevated levels of stress over time, especially in older and unhealthy individuals, is linked to serious health consequences, including a weakened immune system, hypertension, hyperglycaemia, and increased risk for heart attack [3–5]. Additionally, psychosocial stress adversely impacts health behaviours, leading to increased rates of smoking and alcohol consumption, as well decreased rates of physical activity [6,7].

Furthermore, the co-occurrence of psychosocial stressors has been proven to have negative additive effects on health outcomes due to the multiple pathways by which the stressors can operate [8]. The cumulative stress hypothesis postulates that accumulation of psychosocial stressors, or repeated exposure to the same stressor, is particularly harmful to one’s health and may result in greater likelihood of disease, as compared to a single stressor [9]. Thus, it is crucial to capture different sources of psychosocial stressors in order to accurately characterize the overall impact of psychosocial stress on health outcomes. Assessing multiple stressors simultaneously may lend insight into possible mechanisms for how stress affects one’s health.

Although the associations between stress and negative health outcomes have been well-established, less is known about how psychosocial stress impacts the epigenome. Broadly, the epigenome is comprised of biochemical modifications to DNA and its related proteins that regulate gene expression without altering the underlying genetic sequence [10]. Epigenetic mechanisms, such as DNA methylation, can change throughout the life course under certain environmental stimuli, and they may help to explain the biological link between psychosocial stress and adverse health outcomes. To date, there have been a large number of candidate gene and epigenome-wide association studies (EWAS) examining the relationship between individual psychosocial stressors and DNA methylation, including socioeconomic status [11,12], maternal anxiety and depression [13,14], childhood adversity [15,16], and work-related strain [17,18]. However, there have only been a few studies that have examined the relationship between methylation and psychosocial stress defined more broadly, capturing multiple aspects of stress [19–21]. Further, these studies were conducted in relatively small samples, and most were performed in a single ancestry population. Nonetheless, results from these studies showed associations between stress and CpG sites that mapped to genes related to hypertension and heart disease, providing preliminary evidence that long-term psychosocial stress may influence downstream health outcomes through epigenomic changes.

One proposed mechanism by which psychosocial stress affects DNA methylation is through health behaviours [22]. As previously stated, psychosocial stress is known to negatively impact health behaviours [6,7]. There is also strong evidence that these same health behaviours, including smoking, alcohol, and physical activity, lead to significant changes in DNA methylation across the epigenome [23,24]. Thus, health behaviours may mediate the relationship between psychosocial stress and DNA methylation to a certain extent. Although alternative relationships between stress and health behaviours are possible, including the impact that smoking and alcohol use may have on relationship stress or financial strain, it is less likely that health behaviours influence psychosocial stress, particularly chronic stressors across the life course [25]. Further, there may be additional mechanisms by which psychosocial stress affects the epigenome, such as increased inflammatory response [26], elevated cortisol levels [27], and psychiatric health conditions that trigger changes to the brain [28]. Nonetheless, these pathways are not well-established.

In this study, we conducted an epigenome-wide association analysis to identify CpG sites associated with long-term cumulative psychosocial stress in a large sample of older adults, which is critical for understanding the biological consequences of psychosocial stress. For CpG sites associated with psychosocial stress, we next conducted a formal mediation analysis to understand if health behaviours, including smoking, alcohol use, and physical activity, or body mass index (BMI), mediate the relationship between stress and DNA methylation.

## Materials and methods

### Sample

Data are from the Health and Retirement Study (HRS), a nationally representative longitudinal cohort study of individuals over age 50. Information regarding the HRS study design has been described elsewhere [29,30]. In brief, HRS, which began in 1992, consists of biennial interviews that assess metrics of physical and mental health, employment, family characteristics, and wealth. In 2006, HRS added the Psychosocial and Lifestyle Questionnaire, or Leave Behind Questionnaire (LBQ), which is a self-reported psychosocial survey administered to a random 50% of the total HRS sample at each biennial interview [31]. This survey captures information regarding participants’ well-being, lifestyle, and social relationships. In addition, all panel respondents who completed an HRS interview in 2016 were invited to participate in an ancillary study, the Venous Blood Study (VBS, *n* = 9,934) [32]. DNA methylation was measured in a sample of participants who completed the VBS blood draw (*n* = 4,018). This analysis was restricted to 2,689 participants who had data on psychosocial stress (2010 or 2012), all covariates, and DNA methylation (2016). Supplemental Figure 1 provides a flow diagram detailing participant inclusion criteria for our primary analysis.

**Figure 1.**
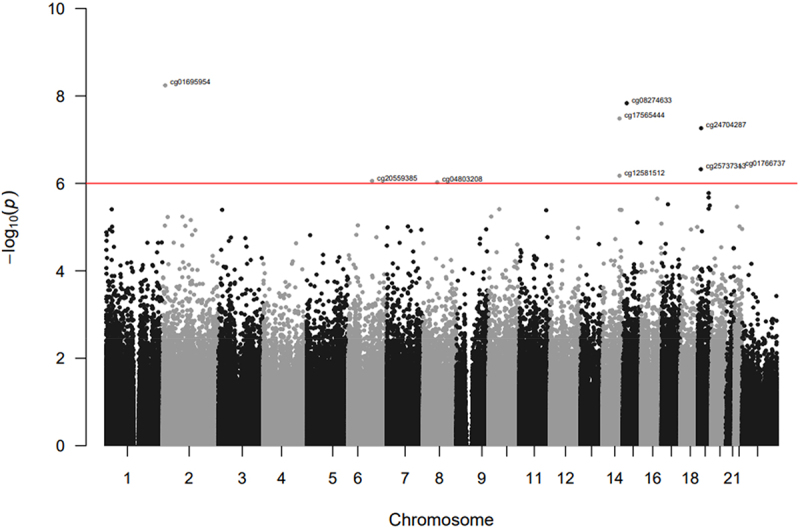
Manhattan plot of the EWAS for cumulative psychosocial stress in HRS. The red horizontal line indicates the epigenome-wide significance threshold (FDR q < 0.10).

To assess the potential impact of restricting our analysis to complete case data, we compared participant characteristics between those who were included (*n* = 2689) and excluded (*n* = 1329) in the primary analysis from the total DNA methylation sample. Student’s t-tests and Chi-squared tests were used, as appropriate. Further, Cohen’s d and Cramer’s V effect sizes were calculated for continuous and categorical variables, respectively, to quantify the size of the difference between the two groups.

### DNA methylation

DNA methylation was assessed from whole blood samples using Illumina Infinium HumanMethylationEPIC Beadchip in a subset of racially and socioeconomically diverse HRS participants. Samples were randomized across plates by key demographic variables, including age, sex, education, and race/ethnicity. The *minfi* R package was used for data preprocessing and quality control [33]. Sex mismatches and probes with an average median intensity < 8.5 were removed, as were probes with missing data for > 5% of the sample [34]. A detection p-value <0.01 was used to remove samples and probes with a detection rate < 5%. Additionally, we removed cross-reactive probes that target repetitive sequences that may map to multiple places in the genome [35]. After quality control, a total of 789,656 CpG in 4,018 samples were available for analysis. At each CpG site, DNA methylation was quantified using beta values, which approximate the proportion of methylation, making them biologically interpretable. To ensure that strong outliers were not driving the results, we winsorized methylation values that were greater than three times the interquartile range (IQR) of the 25^th^ and the 75th percentiles [36,37]. The Houseman Method was used to estimate white blood cell proportions [38].

### Psychosocial stress

The Psychosocial Leave Behind Questionnaire collects data on a variety of psychosocial stress measures [31]. Because this survey was administered to only half of participants at each wave, we utilized data from the 2010 and 2012 interviews to ensure all participants had the opportunity to respond to the psychosocial survey. We examined six psychosocial stress domains, consistent with previous studies: 1) acute life events, 2) financial stress, 3) neighbourhood stress, 4) relationship stress, 5) lifetime discrimination, and 6) childhood adversity [8,39]. Because we are interested in understanding how lifetime stress exposure affects DNA methylation, we examined both acute and chronic measures of stress from childhood throughout adulthood [40]. Each stress domain included one or more stress measures, as described below and in Supplemental Table 1. For a measure to be considered complete, participants had to have answered at least 80% of the questions within the measure, unless otherwise noted in the measurement scoring instructions [41]. Only participants with completed stress measures were included in the analysis. We transformed each stress measure into a z-score. For domains that included multiple stress measures, we summed the individual z-scores together and then standardized the summed score into a z-score to allow for cross-domain comparisons [42,43].

**Table 1.** Characteristics of the health and retirement study analytic sample (*N* = 2,689).

Characteristics	N (%) or mean (SD)
Age, years	70.4 (9.5)
Female	1590 (59.1)
Race/Ethnicity	
Hispanic	272 (10.1)
Black	350 (13.0)
White	1997 (74.3)
Other	69 (2.6)
Education	
No degree	346 (12.9)
HS degree	1631 (60.7)
College degree or higher	712 (26.5)
Employment Status	
Working for pay	1175 (43.7)
Not working for pay	1514 (56.3)
Marital Status	
Married/Partnered	1905 (70.8)
Single/Widowed/Divorced	784 (29.2)
Have Children	2390 (88.9)
Total Household Wealth ($)	442,619 (952,905)
Smoking Status	
Never smoker	1208 (45.2)
Former smoker	1138 (42.6)
Current smoker	327 (12.2)
Alcohol Use	
Never drinker	1588 (59.1)
Moderate drinker	914 (34.0)
Heavy drinker	186 (6.9)
Physical Activity	
Active	1591 (59.2)
Inactive	1096 (40.8)
BMI (kg/m^2^)	30.4 (6.7)

In total, we assessed six stress domains. *Acute life events* included two measures: (1) acute lifetime traumas (seven items) and (2) acute stressful life events in the past 5 years (six items). *Financial stress* included two measures: (1) financial strain (two items) and 2) lack of financial autonomy (two items). *Neighborhood stress* included one measure (four items), focusing on neighbourhood safety, vandalism, and cleanliness. *Relationship stress* included four measures: (1) spouses (four items), (2) children (four items), (3) other family members (four items), and friends (four items). *Lifetime discrimination*, which measures racial and nonracial discrimination, included two measures: (1) perceived everyday discrimination (five items) and (2) major discriminatory events (six items). *Childhood adversity* included one measure related to lifetime traumas in adolescence (four items).

Using the six stress domains, we created a cumulative psychosocial stress score by summing the standardized scores from the six stress domains and then re-standardizing the summed scores [8]. This cumulative stress score represents the total stress exposure across all stress domains, and it is the main exposure of interest in our analyses. Furthermore, because the effects of childhood adversity may operate differently on the epigenome than the other five stress domains measured in adulthood, we created an additional variable to represent adult psychosocial stress by summing the standardized score from the five stress domains measured in adulthood (acute life events, neighbourhood stress, financial stress relationship stress, and lifetime discrimination) and re-standardizing the summed score [44]. This allowed us to compare results when assessing cumulative stress from adulthood versus throughout the life course.

### Covariates

Sociodemographic factors were collected at the 2010 or 2012 interview, concurrent with the timing of the Psychosocial Leave Behind Questionnaire. Consistent with previous literature, we included the following variables in our adjusted regression models: age, sex (male or female), educational attainment (no degree, high school degree, college degree or higher), employment status (yes/no), marital status (married/partnered or single/widowed/divorced), having any children (yes/no), and self-reported total household wealth in US dollars [8,39–42]. Total wealth was calculated as the sum of all assets (stocks, mutual funds, investments, bonds, checking and savings accounts, and all other savings) minus all debt [41,42]. To avoid large standard errors, we log-transformed total household wealth using the modulus transformation, sign(total wealth)*log(|total wealth|+1) [45]. The top 10 principal components (PCs) of genetic ancestry were estimated using genome-wide genotype data and were used as model covariates to account for population stratification and admixture [46].

### Potential mediators

Like sociodemographic factors, health behaviours, including smoking (never, former, current), alcohol use (never, moderate, heavy), and moderate or vigorous physical activity (active, inactive), and BMI were collected at the 2010 or 2012 interview and were considered potential mediators of the association between psychosocial stress and DNA methylation. The same set of variables was also collected on a subset of participants who had available data in 2014 (*n* = 2,614). Based on the National Institute on Alcohol Abuse and Alcoholism criteria, moderate drinking was defined as 1–7 drinks per week for females and 1–14 drinks per week for males, while heavy drinking was defined as > 7 drinks per week for females and > 14 drinks per week for males [47]. Physical activity was calculated based on the self-reported frequencies of moderate or vigorous activities. Participants reporting that they engaged in moderate or vigorous physical activity at least once per week were classified as active, while those reporting physical activity less than once per week were classified as inactive [48]. BMI was calculated from participant’s weight in kilograms divided by the square of their height in metres, measured during the physical health section of their interview [49]. For participants who did not have their height and weight measured during their interview, we used their self-reported BMI. We performed Spearman’s rank-order correlations to assess the strength of associations between health behaviours. Physical activity, alcohol use, and smoking status were analysed as ordinal variables.

### Epigenome-wide association analysis

We conducted an epigenome-wide association study (EWAS) of cumulative psychosocial stress using multivariable linear regression to estimate associations of stress with DNA methylation at each CpG site, adjusting for potential confounders, including age, sex, educational attainment, top 10 genetic ancestry PCs, year of psychosocial stress measurement, white blood cell proportions, and technical covariates, including sample plate, plate row, and plate column (Model 1). To correct for multiple testing, the Benjamini-Hochberg procedure was applied to control the false discovery rate (FDR) at a threshold of q < 0.10. The minimally adjusted multivariable model (Model 1) was considered our primary model. We investigated the attenuation of effects on all significant CpG sites identified in our primary model by further adjusting for marital status, employment status, total income, and having children (Model 2).

As a sensitivity analysis, we conducted an EWAS of the psychosocial stress score comprised only of the five stress domains measured in adulthood (i.e., excluding childhood adversity) using Model 1. This allowed us to understand if the time period where stress was experienced (adulthood vs. life course) influenced the associations between stress and DNA methylation.

Next, to assess how much each of the six stress domains were driving the association between cumulative psychosocial stress and DNA methylation, we examined the associations of each stress domain separately with the significant CpG sites identified in the primary analysis (Model 1). Analyses were conducted using R software (version 4.1.3).

### Mediation analysis

To investigate the mediating effect of health behaviours on the relationship between psychosocial stress and DNA methylation, we conducted a formal mediation analysis for all significant CpG sites identified in the EWAS of cumulative stress (Model 1) using the Karlson-Holm-Breen (KHB) method [50]. The KHB method, which has a structural equation modelling (SEM) framework, decomposes the total effect of an independent variable into the sum of the direct and indirect effects [51]. Although this method was primarily developed for non-linear models, it is also appropriate for linear models [52]. A major advantage of KHB, as opposed to other mediation methods, is that it provides unbiased estimates for the overall, direct, and indirect effects on the same scale, thus achieving reliable comparisons across different mediators of different variable types [53]. Additionally, this method allows for the examination of multiple mediators simultaneously and has the ability to disentangle the effects of correlated mediators, thereby quantifying the relative contribution of each mediator to the total effect [54,55].

We used the *khb* R package to test mediation by smoking, alcohol use, physical activity, and BMI simultaneously [50]. Due to computational constraints of KHB, we pre-adjusted DNA methylation for white blood cell proportions and batch effects using linear models (sample plate, plate row, plate column), and we used the residuals as the outcome in the mediation models. For each CpG site, we conducted a generalized linear model (GLM) following a Gaussian (normal) distribution [51,52]. All mediation models were adjusted for age, sex, educational attainment, employment status, marital status, having any children, total income, genetic ancestry PCs, and year of psychosocial stress measurement, consistent with the adjustment variables in Model 2 of our EWAS. In the mediation analysis, both psychosocial stress and health behaviours were collected at least four years prior to DNA methylation, thereby reducing the concern for reverse causation, where DNA methylation could influence either stress or health behaviours. Additionally, we conducted a secondary mediation analysis on all significant CpG sites identified in the EWAS of cumulative psychosocial stress for participants who had available health behaviour and BMI data in 2014 (*n* = 2,614). In this sensitivity analysis, the potential mediators were collected after psychosocial stress, as opposed to concurrently, further establishing temporality between psychosocial stress and health behaviours.

### Enrichment of genomic features

For CpG sites that were significantly associated with psychosocial stress, we examined whether their genomic locations were enriched for features, including gene promoters, enhancers, DNase I hypersensitivity sites (DHS), CpG islands, CpG island flanking shores, or CpG island flanking shelves. We considered a CpG site to be in the promoter region if it was located less than 1.5 kb upstream of a transcriptional start site. A CpG site was designated as a CpG island (CGI) flanking shore or shelf if it was within 2 kb or between 2–4 kb of a CGI, respectively. Annotation files from Illumina and the UCSC genome browser were used to identify the target genes and genomic features associated with each CpG [56,57]. Enrichment analysis was performed using a two-sided Fisher’s exact test.

## Results

### Descriptive statistics

Sample characteristics are shown in Table 1. The mean age of participants was 70.4 years (SD: 9.5), the majority were female (59%), and a quarter of participants had a college degree or higher. Overall, 74% of participants were non-Hispanic white, 13% were non-Hispanic Black, 10% were Hispanic, and 3% were another race or ethnicity. Slightly less than half of the participants were working for pay at the time the psychosocial battery was administered (43.7%). Approximately 70% of participants were either married or had a partner, while the remaining 30% were either single, widowed, or divorced. Additionally, 89% had at least one child. Average household wealth for participants was $442,619 (SD: 952,905). In terms of participants’ health behaviours, 10% were current smokers, 40% were moderate to heavy drinkers, and 59% participated in moderate or vigorous exercise at least once per week. The mean measured BMI for participants was 30.4 kg/m^2^ (SD: 6.7). Participants from the DNA methylation sample with complete data on stress and covariates tended to be older, have higher education, and have healthier behaviours than those who were excluded, although the effect sizes between the two groups were relatively small (Cramer’s V and Cohen’s d < 0.30 for all covariates) (Supplemental Table S2).

All six of the psychosocial stress domains were correlated with one another at *p* < 0.001 (Supplemental Table S3). The correlation coefficients ranged from 0.10 to 0.39, with the highest measures of correlation occurring between lifetime discrimination and relationship stress, as well as lifetime discrimination and financial stress. Health behaviours and BMI were weakly, but significantly, correlated with one another (Supplemental Figure S2). BMI and physical activity had the strongest correlation (Spearman’s =-0.19, *p* < 0.001).

### Epigenome-wide association analysis

**Figure 2.**
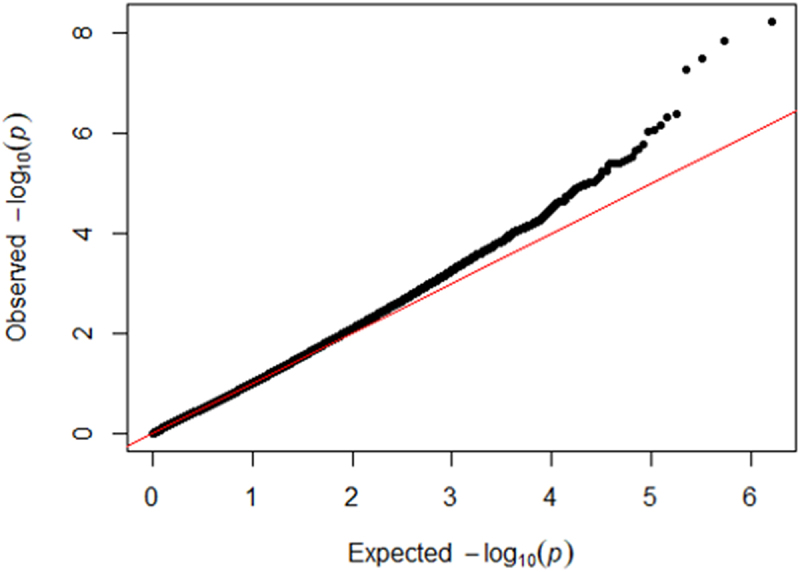
Quantile-quantile plot of the EWAS for cumulative psychosocial stress in HRS (inflation factor λ = 1.09).

In the primary EWAS (Model 1), we identified nine CpG sites associated with psychosocial stress (FDR q < 0.10, all *p* < 9E–07; Figures 1 and 2). Eight of the CpGs were less methylated with higher levels of cumulative stress. When adding additional sociodemographic characteristics that may be on the causal pathway from early life stress exposure to later life DNA methylation to the primary model (Model 2), effect estimates of the nine CpG sites were slightly attenuated but remained at least nominally associated with psychosocial stress (all *p* < 2E–05).

In the sensitivity analysis that excluded childhood adversity from the cumulative psychosocial stress score, we observed very similar results to those obtained from our original EWAS in Model 1 (Supplemental Table S4). Five CpG sites were associated with cumulative psychosocial stress in adulthood (FDR q < 0.10), and those CpG sites matched the top five results from the cumulative stress EWAS in Model 1. Furthermore, the remaining four CpG sites that were significant in our original EWAS were all at least nominally associated with cumulative psychosocial stress in adulthood (all *p* < 9E–06).

When assessing the relationship between the six individual stress domains and the nine CpG sites, we found that acute life events, financial stress, and lifetime discrimination domains had at least nominally significant associations with all CpG sites (*p* < 0.05), while neighbourhood stress, relationship stress, and childhood adversity each had nominal associations with at least six CpG sites (Supplemental Table S5). These results indicate that the associations between DNA methylation and the domains of stress that contributed to the cumulative psychosocial stress score were relatively consistent.

### Mediation analysis

We next investigated the mediating effect of health behaviours on the association between psychosocial stress and DNA methylation. Cumulative psychosocial stress explained between 0.6% and 4.8% of the total variability of the nine identified CpG sites in our primary model (Model 1) and between 0.5% and 4.5% of the total variability after adjusting for additional sociodemographic covariates (Model 2; Supplemental Table S6). When assessed simultaneously, health behaviours (smoking, alcohol use, physical activity) and BMI significantly mediated 9.4% to 21.8% of the relationship between stress and methylation at eight of the nine CpG sites (Supplemental Table S7). Specifically, current smoking mediated between 5.4% and 8.8% of the total effect of psychosocial stress on DNA methylation at four CpG sites (cg17565444, cg01695954, cg01766737, cg12581512; all *p* < 0.05). Moderate alcohol use mediated 3.9% of the total effect in cg04803208 (*p* = 0.04). Finally, between 4.0% and 10.4% of the total effect of psychosocial stress on DNA methylation was mediated by BMI at seven CpGs (cg25737313, cg08274633, cg01695954, cg24704287, cg01766737, cg12581512, cg04803208; all *p* < 0.05). Physical activity did not significantly mediate the relationship between stress and methylation at any of the nine CpG sites. It should be noted that the total effects in these mediation models were substantively similar to those in the Model 2 EWAS (Table 2). Any slight differences observed between the two sets of analyses are due to the pre-adjustment of methylation beta values for batch effects and white blood cell proportions prior to conducting the mediation analysis.

**Table 2.** Significant CpG sites from the epigenome-wide association study (EWAS) of cumulative psychosocial stress (Model 1: FDR q < 0.10).

CpG Site	Functional Annotation				Model 1			Model 2	
	*Chr*	*Gene*	*Relation to CpG Site*	*Genomic Feature*	*Beta*	*P-value*	FDR q	*Beta*	*P-value*
cg01695954	2	*RRM2*	S_Shore	Promoter	−0.40	5.7E–09	0.0045	−0.38	8.4E–08
cg08274633	15	*C15orf53*	OpenSea	Promoter	−0.42	1.5E–08	0.0058	−0.42	4.4E–08
cg17565444	14	*ASB2*	OpenSea	Body	−0.32	3.3E–08	0.0086	−0.30	4.8E–07
cg24704287	19	*MIR23AHG*	N_Shore	Body	−0.66	5.5E–08	0.011	−0.59	2.9E–06
cg01766737	22	*CENPM*	N_Shore	Body	−0.13	4.2E–07	0.063	−0.11	1.1E–05
cg25737313	19	*HOOK2*	N_Shore	Body	−0.28	4.7E–07	0.063	−0.27	2.8E–06
cg12581512	14	*ASB2*	OpenSea	Body	−0.29	6.7E–07	0.075	−0.27	8.2E–06
cg20559385	6		OpenSea		−0.61	8.8E–07	0.083	−0.67	1.6E–07
cg04803208	8	*LOC100130298*	OpenSea	Body	0.51	9.4E–07	0.083	0.51	2.1E–06

Beta is the regression coefficient representing the percent change in DNA methylation associated with a 1 standard deviation increase in the cumulative psychosocial stress score

Model 1: CpG Site ~ Psychosocial Stress Score + Age + Sex + Educational Attainment + Interview Year + Genetic Ancestry PC 1-10 + White Blood Cell Proportions + Row Position on Chip + Column Position on Chip + Sample Plate

Model 2: Model 1 + Marital Status + Employment + Has Children + Total Household Wealth Associations were only evaluated in Model 2 if they had an FDR q <0.10 in Model 1.

In the sensitivity analysis testing for mediation by health behaviours collected in 2014, we observed similar results to our primary mediation analysis (Supplemental Table S8). Health behaviours and BMI significantly mediated 10.1% to 26.0% of the relationship between stress and methylation at all nine CpG sites, with similar contributions from smoking, alcohol, and BMI. One difference between the two analyses was the impact of physical activity on the relationship between stress and methylation. When physical activity was collected in 2014, it mediated between 3.8% and 5.1% of the total effect of stress on DNA methylation at four CpGs (cg01695954, cg24704287, cg12581512, cg20559385; all *p* < 0.05), which is slightly more than observed in the primary mediation model for those CpG sites (0.7%-2.1%).

### Genomic feature enrichment analysis

Using the Illumina annotation gene mapping, we found that CpGs associated with psychosocial stress (FDR q < 0.10) were enriched for enhancers (OR:8.38, *p* = 0.034), but not for promoters, DHS, CGIs, or CGI flanking shores or shelves.

## Discussion

This large-scale EWAS of long-term psychosocial stress in whole blood samples from a multi-ancestry cohort of older adults identified nine CpG sites. We found that health behaviours and BMI may partially mediate the relationship between psychosocial stress and DNA methylation; however, given that these factors explained a relatively low proportion of the relationship, it is likely that stress also impacts methylation through mechanistic pathways independent of these factors.

Our most significant CpGs associated with psychosocial stress, cg01695954, is in the promoter region of the ribonucleotide reductase regulatory subunit M2 (*RRM2*) gene on chromosome 2. This gene encodes one of two non-identical subunits for ribonucleotide reductase (RNR), which is important for DNA synthesis and DNA repair by producing deoxynucleoside triphosphate (dNTP) [58]. Additionally, its expression is regulated by both transcription and protein degradation. Studies have found that *RRM2* plays a crucial role in cell death, affecting autoimmune disorders such as rheumatoid arthritis (RA), as well as several types of cancer [59]. [60]. In particular, the inhibition of *RRM2* results in a decrease in cellular proliferation and in increase in apoptosis, thus representing a potential chemotherapeutic target and treatment for RA. DNA methylation in or near *RRM2* has also been associated with schizophrenia and post-traumatic stress disorder (PTSD), two psychosocial disorders related to psychosocial stress [61,62]. In an EWAS analysis, PTSD was associated with methylation of cg18623836, a CpG site located in the promoter region of RRM2, 112 bps from our significant CpG, cg01695954 [61]. This finding supports the hypothesis that the epigenetic patterning of the *RRM2* genomic region may be impacted by long-term psychosocial distress.

Another CpG associated with psychosocial stress in our study is cg08274633, located in the promoter region of an uncharacterized gene, *C15orf53*. This CpG has been associated with ageing [63], clear cell renal carcinoma [64], incident COPD [65], and incident type 2 diabetes [65] in previous large-scale EWAS. The observed directions of cg08274633 methylation on COPD and type 2 diabetes indicate a potential pathway where elevated psychosocial stress results in decreased DNA methylation at cg08274633, which is in turn associated with increased risk of COPD and type 2 diabetes. Future studies can examine this pathway in greater detail to understand the underlying mechanisms linking stress, DNA methylation, and chronic diseases.

Two identified CpG sites, cg17565444 and cg12581512, map to the gene body of the ankyrin repeat and SOCS box containing 2 *(ASB2*) gene on chromosome 14. This gene has been associated with BMI [66], smoking [67], waist circumference [68], cognition [69], inflammation [70], and HIV infection [71] *ASB2* encodes a protein that plays a crucial role in protein degradation by coupling cytokine signalling protein suppressors with the elongin BC complex [72]. Additionally, this gene plays a role in growth inhibition induced by retinoic acid, a metabolite of vitamin A that is essential for embryogenesis and the regulation of regeneration and plasticity in the adult brain [73].

Several EWAS have failed to detect significant associations between psychosocial stressors and DNA methylation, and those that detected associations had relatively poor consistency across studies [74]. This may be due to challenges faced when conducting EWAS of psychosocial stress, including small sample sizes, lack of appropriate adjustment covariates, and inconsistent methods for measuring stress. Kalinowski et al.. (2022), which examined the relationship between stress overload and DNA methylation among 228 African American women, did not identify any CpGs at the epigenome-wide significance threshold, likely due to the small sample size; however, the top CpGs identified mapped to genes related to heart disease and hypertension [19]. Lam et al.. (2012) found associations between DNA methylation and several stress measures including socioeconomic status, perceived stress, and cortisol levels, in a community cohort of 92 individuals, but this analysis did not adjust for any potential confounders [20]. Our study aimed to address these challenges by conducting an EWAS in a diverse population of 2,689 older adults, where we captured both a comprehensive set of objective and subjective stress measures across the life course and adjusted for relevant potential confounders. In a recent systematic review investigating DNA methylation signatures of broad psychosocial stressors (Zhang et al.. (2022)), none of our findings overlapped with those from the candidate gene studies and EWAS identified in the review, likely due to the reasons stated above [74].

Health behaviours and BMI explained a relatively modest, but significant, percentage of the relationship between stress and DNA methylation. Cumulatively, smoking, alcohol consumption, physical activity, and BMI mediated between 9% and 22% of the relationship between stress and methylation at the nine CpG sites identified in the EWAS. Further, we achieved similar results in a sensitivity analysis using a subset of participants who had health behaviour and BMI data measured in 2014. One explanation is that we may not have fully captured the mediating effect of health behaviours on the relationship between stress and DNA methylation. The health behaviours we assessed as potential mediators were only measured at a single timepoint. Because this is a population of older adults, health behaviours from earlier life, which were not captured in our analysis, could have mediated the association between stress and DNA methylation. Furthermore, the health behaviours selected for mediation testing may not have been comprehensive. Other health behaviours, aside from those included in our analysis, could be potential mediators on the relationship between stress and DNA methylation. Alternatively, psychosocial stress may affect DNA methylation through additional underlying pathways independent of health behaviours.

One pathway through which long-term psychosocial stress may influence DNA methylation is through increased levels of cortisol. When a stressor is perceived and interpreted, the stress response system, or the hypothalamic-pituitary-adrenal axis (HPA axis), is activated [75]. The hypothalamus area of the brain sends a chemical message to the pituitary glands, which triggers the release of adrenocorticotropic hormone (ACTH). In turn, ACTH stimulates the adrenal glands to release cortisol, a steroid hormone that binds glucocorticoid receptors. Because glucocorticoid receptors are found in nearly every tissue of the body, once cortisol attaches to its receptors, it interacts with many organ systems, including the nervous, immune, respiratory, and cardiovascular systems [76]. After the stressor has been resolved, built-in regulators help return the psychological systems to usual levels, restoring the body to homoeostasis. However, chronic stress can lead to long-term activation of the stress response system, resulting in elevated levels of cortisol and other stress hormones, which may disrupt the development of brain architecture and other organ systems, potentially leading to changes in DNA methylation. Glad et al.. (2017) found that excessive cortisol exposure over time prompted changes in global DNA methylation patterns, particularly in genes known to induce hyperactivity of the HPA-axis [77].

Another mechanism by which psychosocial stress may affect DNA methylation is through psychiatric health conditions. Long-term psychosocial stress may trigger certain psychiatric disorders, including anxiety and depression, which in turn may affect DNA methylation through alterations to brain anatomy [78,79]. Neuroimaging studies of individuals with major depressive disorder (MDD) found significant alterations to brain regions, including the frontal lobe, hippocampus, parietal lobe, striatum, and thalamus, as well as impairment of crucial circuits in the brain [78]. For example, the prefrontal-subcortical circuit, which is comprised of the striatum, thalamus, and prefrontal cortex and is involved heavily in emotional and cognitive processing, may be impaired in individuals with MDD, leading to emotional and cognitive dysfunction. Furthermore, a prior EWAS found that MDD was associated with differently methylated CpG sites through mechanisms involved in neuronal synaptic plasticity and calcium signalling, which are involved in the neurobiology of depressive disorders [79].

As with any study, there are limitations. First, due to the novel research question being studied, we were unable to replicate our findings in an independent cohort. Additionally, we only used self-reported psychosocial stress data to create the cumulative stress score. Future analyses can utilize multiple sources of stress, including changes in physiological markers and behaviours, to get a more comprehensive definition of cumulative stress. Furthermore, we weighed all six psychosocial stress domains equally when calculating cumulative stress score, but certain stress domains may have a greater impact on DNA methylation than others.

Although we implemented a longitudinal study design, we cannot rule out the possibility of reverse causality due to the small timeframe between measurement of the exposure, mediators, and outcome variables. For future analyses, it would be useful to measure both DNA methylation and psychosocial stress at multiple time points to assess changes in the epigenome in response to stress. Additionally, we only examined the mediating role of BMI on the association between stress and DNA methylation, although it is possible that other relationships exist. For example, BMI may be a downstream consequence of methylation changes. Future research should further explore whether BMI, as well as other comorbidities, lie in the causal pathway between stress and DNA methylation or are subsequent to methylation changes.

Since data were collected from a cohort of older adults, there is the possibility of selection bias in our study. Participants who had severe life stressors may have been less likely to participate or remain in the cohort. Thus, the psychosocial stress scores that we calculated based on our sample may differ slightly from what we would observe in the general U.S. population, although the literature is mixed on whether psychosocial distress and mental health influence study participation or attrition [80–83]. Finally, because health behaviours were measured concurrently with psychosocial stress, the associations between stress and certain health behaviours may have been bi-directional. Consequently, in addition to mediators, these health behaviours could have also been confounders on the relationship between stress and DNA methylation. To help establish a temporal relationship between the exposure (stress) and mediators (methylation), we conducted a sensitivity mediation analysis using health behaviours and BMI measured in 2014, two to four years after psychosocial stress measurement, and the results were substantively similar to our primary analysis.

This study also has several key strengths. First, we examined psychosocial stress in both childhood and adulthood, which allowed us to better understand the impact of cumulative stress on DNA methylation across the life course, not just at a specific period in time. Furthermore, we examined the stress domains in sensitivity analyses. We found that although there were similarities among the domains, certain stress domains were driving the associations between cumulative stress and DNA methylation more than others. Finally, we conducted our research in a multi-ancestry population, including African, Hispanic/Latino, and European ancestries, allowing us to study the effects of cumulative stress in a diverse population of older adults.

In summary, our results support the hypothesis that cumulative psychosocial stress is associated with a subset of CpG sites in the epigenome. Additionally, health behaviours mediate only a small percentage of this relationship, providing evidence that independent mechanisms may link psychosocial stress and DNA methylation. Future studies are needed to replicate our findings in an independent cohort in order to confirm the observed associations between psychosocial stress and DNA methylation.

## Supplementary Material

Opsasnick StressEWAS SuppTables Revised.docx

## Data Availability

The data used in this study are available from the Health and Retirement Study (HRS), conducted at the University of Michigan. These data are available to registered users who meet security requirements and agree to data use conditions specified by the HRS (https://hrsdata.isr.umich.edu/data−products/).
