# Supplementary material for: Epigenome-wide association study of long-term psychosocial stress in older adults
Source: Epigenetics. 2024 Mar 3;19(1):2323907. doi: 10.1080/15592294.2024.2323907 (PMC10913704; doi:10.1080/15592294.2024.2323907)
Supplement: Opsasnick StressEWAS SuppTables Revised.docx [file KEPI_A_2323907_SM1076.docx]

Supplemental Figure 1: Flow diagram detailing participant inclusion criteria in primary analysis.


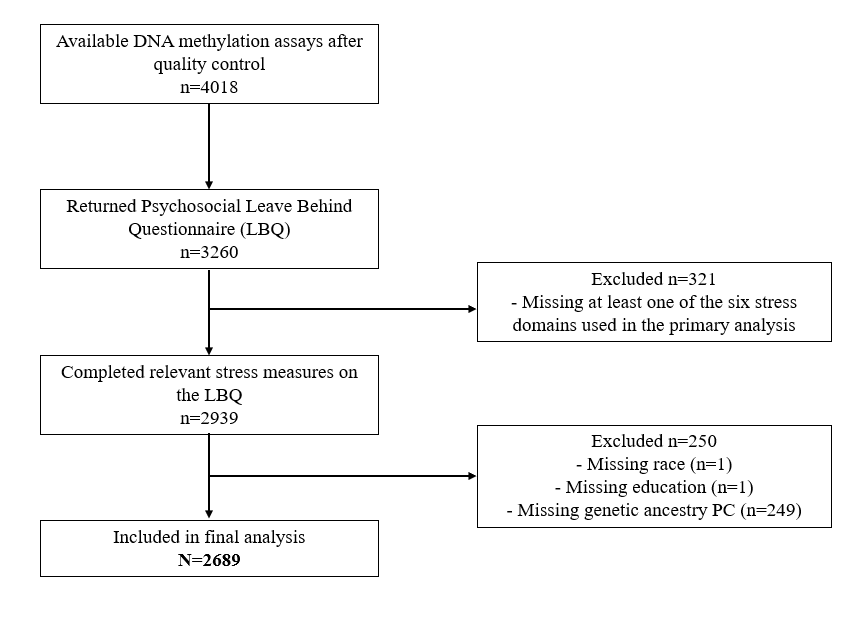


| Supplemental Table 1: Psychosocial stress measures included in the 6 stress domains. | | |
| --- | --- | --- |
| **Stress Domain** | **Stress Measure** | **Individual Items** |
| **Acute Life Events** | Lifetime Traumas | (1) Has experienced the death of child; (2) has ever been in a major fire, flood, earthquake or other natural disaster; (3) has ever fired a weapon in combat or been fired upon in combat; (4) has a spouse, partner, or child has ever been addicted to drugs or alcohol; (5) was a victim of a serious physical attack or assault; (6) has ever had a life-threatening illness or accident; (7) has spouse or a child has ever had a life-threatening illness or accident. |
|  | Stressful Life Events in Past 5 Years | (1) Has involuntarily lost a job for reasons other than retirement; (2) has been unemployed and looking for work for longer than 3 months; (3) was anyone in household unemployed and looking for work for longer than 3 months; (4) have moved to a worse residence or neighborhood; (5) have been robbed or home has been burglarized; (6) has been victim of fraud. |
| **Financial Stress** | Financial Strain | (1) Difficulty meeting monthly payments on bills; (2) distress caused by ongoing financial strain. |
|  | Lack of Financial Autonomy | (1) Satisfaction with his/her current financial situation; (2) amount of control over his/her financial situation (*reverse-coded*). |
| **Neighborhood Stress** | Neighborhood Disorder | (1) Vandalism and graffiti are a big problem in this area; (2) People would be afraid to walk alone in this area after dark; (3) This area is always full of rubbish and litter; (4) There are many vacant or deserted houses or storefronts in this area. |
| **Relationship Stress** | Marital Stressors | (1) How often do they make too many demands on you? (2) How much do they criticize you? (3) How much do they let you down when you are counting on them? (4) How much do they get on your nerves? |
|  | Child-Related Stressors |  |
|  | Other Family-Related |  |
|  | Friend Stressors |  |
| **Lifetime Discrimination** | Major Discriminatory Events | (1) Have been unfairly dismissed from a job; (2) have been unfairly not hired for a job; (3) have been unfairly denied a promotion; (4) have been unfairly prevented from moving into a neighborhood because the landlord or a realtor refused to sell or rent you a house or apartment; (5) have been unfairly denied a bank loan; (6) have been unfairly stopped, searched, questioned, physically threatened or abused by the police. |
|  | Perceived Everyday Discrimination | (1) Have been treated with less courtesy or respect than other people; (2) have received poorer service than other people at restaurants or stores; (3) people act as if they think you are not smart; (4) people act as if they are afraid of you; (5) you are threatened or harassed. |
| **Childhood Adversity** | Childhood Adversity Before Age 18 | (1) Had to do a year of school over again; (2) was ever in trouble with the police; (3) either of his/her parents drank or used drugs so often that it caused problems in the family; (4) was ever physically abused by his/her parents. |

| Supplemental Table 2: Participant characteristics comparing those included and excluded in the primary analysis from the full DNA methylation sample (N=4018) |
| --- |

| **Characteristics** | **Included (n=2689)**  **N(%) or mean (SD)** | **Excluded (n=1329)**  **N(%) or mean (SD)** | **Effect Size**  **(Cohen’s d or Cramer’s V)** | **P-value** |
| --- | --- | --- | --- | --- |
| Age, years | 70.4 (9.5) | 67.5 (9.6) | 0.30 | <0.001 |
| Female | 1590 (59.1) | 759 (57.1) | 0.02 | 0.22 |
| Race/Ethnicity |  |  | 0.24 |  |
| Hispanic | 272 (10.1) | 295 (22.2) |  | <0.001 |
| Black | 350 (13.0) | 308 (23.2) |  |  |
| White | 1997 (74.3) | 672 (50.6) |  |  |
| Other | 69 (2.6) | 53 (4.0) |  |  |
| Education |  |  | 0.16 |  |
| No degree | 346 (12.9) | 329 (24.8) |  | <0.001 |
| HS degree | 1631 (60.7) | 744 (56.0) |  |  |
| College degree or higher | 712 (26.5) | 255 (19.2) |  |  |
| Employment Status |  |  | 0.26 |  |
| Working for pay | 1175 (43.7) | 917 (71.5) |  | <0.001 |
| Not working for pay | 1514 (56.3) | 365 (28.5) |  |  |
| Marital Status |  |  | 0.08 |  |
| Married/Partnered | 1905 (70.8) | 812 (63.2) |  | <0.001 |
| Single/Widowed/Divorced | 784 (29.2) | 473 (36.8) |  |  |
| Have Children | 2390 (88.9) | 1170 (88.0) | 0.08 | 0.41 |
| Total Household Wealth ($) | 442,619 (952,905) | 304,965 (795,877) | 0.15 | <0.001 |
| Smoking Status |  |  | 0.07 |  |
| Never smoker | 1208 (45.2) | 539 (42.1) |  | <0.001 |
| Former smoker | 1138 (42.6) | 523 (40.9) |  |  |
| Current smoker | 327 (12.2) | 218 (17.0) |  |  |
| Alcohol Use |  |  | 0.06 |  |
| Never drinker | 1588 (59.1) | 826 (64.4) |  | 0.002 |
| Moderate drinker | 914 (34.0) | 365 (28.5) |  |  |
| Heavy drinker | 186 (6.9) | 91 (7.1) |  |  |
| Physical Activity |  |  | 0.05 |  |
| Active | 1591 (59.2) | 692 (53.8) |  | 0.001 |
| Inactive | 1096 (40.8) | 594 (46.2) |  |  |
| BMI (kg/m^2^) | 30.4 (6.7) | 29.2 (6.5) | 0.19 | <0.001 |

| Supplemental Table 3: Pearson correlation coefficients between the 6 psychosocial stress domains. | | | | | | |
| --- | --- | --- | --- | --- | --- | --- |
|  | **Acute Life Events** | **Financial Stress** | **Neighborhood Stress** | **Relationship Stress** | **Lifetime Discrimination** | **Childhood Adversity** |
| **Acute Life Events** | 1.00 | 0.33 | 0.11 | 0.21 | 0.35 | 0.20 |
| **Financial Stress** | 0.33 | 1.00 | 0.24 | 0.35 | 0.37 | 0.14 |
| **Neighborhood Stress** | 0.11 | 0.24 | 1.00 | 0.21 | 0.21 | 0.10 |
| **Relationship Stress** | 0.21 | 0.35 | 0.21 | 1.00 | 0.39 | 0.15 |
| **Lifetime Discrimination** | 0.35 | 0.37 | 0.21 | 0.39 | 1.00 | 0.20 |
| **Childhood Adversity** | 0.20 | 0.14 | 0.10 | 0.15 | 0.20 | 1.00 |
| All correlations between pairs of stress domains had p<0.001. | | | |  |  |  |

Supplemental Figure 2: Heat map of Spearman correlations between health behaviors and BMI.
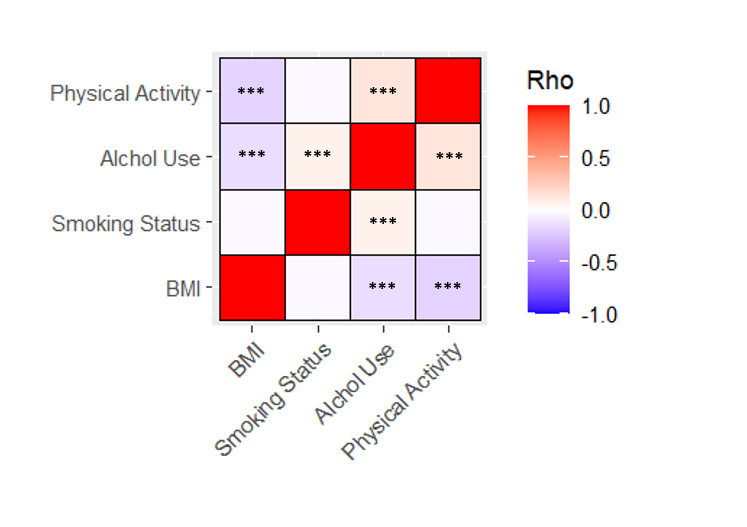


***p<0.001; all other correlations had p>0.10.

Supplemental Table 4: Association between adult psychosocial stress and the 9 CpG sites that were significant in cumulative psychosocial stress EWAS (Model 1; FDR q <0.10)

| **CpG Site** | **Functional Annotation** | | | |  | |  |
| --- | --- | --- | --- | --- | --- | --- | --- |
|  |  |  |  |  |  | |  |
|  | *Chr* | *Gene* | *Relation to CpG Site* | *Genomic Feature* | *Beta* | *P-value* | *FDR q* |
| cg01695954 | 2 | *RRM2* | S_Shore | Promoter | -0.42 | 8.0E-10 | 0.00063 |
| cg08274633 | 15 | *C15orf53* | OpenSea | Promoter | -0.37 | 3.4E-07 | 0.076 |
| cg17565444 | 14 | *ASB2* | OpenSea | Body | -0.29 | 3.8E-07 | 0.076 |
| cg24704287 | 19 | *MIR23AHG* | N_Shore | Body | -0.63 | 2.1E-07 | 0.076 |
| cg01766737 | 22 | *CENPM* | N_Shore | Body | -0.12 | 5.9E-07 | 0.093 |
| cg25737313 | 19 | *HOOK2* | N_Shore | Body | -0.25 | 3.5E-06 | 0.26 |
| cg12581512 | 14 | *ASB2* | OpenSea | Body | -0.25 | 9.2E-06 | 0.28 |
| cg20559385 | 6 |  | OpenSea |  | -0.58 | 2.1E-06 | 0.22 |
| cg04803208 | 8 | *LOC100130298* | OpenSea | Body | 0.49 | 2.2E-06 | 0.22 |

Beta is the regression coefficient representing the percent change in DNA methylation associated with a 1 standard deviation increase in the adult psychosocial stress score.

Model: CpG Site ~ Adult Psychosocial Stress Score + Age + Sex + Educational Attainment + Interview Year + Genetic Ancestry PC 1-10 + White Blood Cell Proportions + Row Position on Chip + Column Position on Chip + Sample Plate

| Supplemental Table 5: Association between the six stress domains and the and the 9 CpG sites that were significant in cumulative psychosocial stress EWAS (Model 1; FDR q <0.10) | | | | | | | | | | | | | | |
| --- | --- | --- | --- | --- | --- | --- | --- | --- | --- | --- | --- | --- | --- | --- |
|  | **Cumulative  Stress** | | **Acute Life Events** | | **Financial  Stress** | | **Neighborhood Stress** | | **Relationship Stress** | | **Lifetime Discrimination** | | **Childhood Adversity** | |
| **CpG Sites** | Beta | P-value | Beta | P-value | Beta | P-value | Beta | P-value | Beta | P-value | Beta | P-value | Beta | P-value |
| cg17565444 | -0.32 | 3.3E-08 | -0.32 | 1.3E-09 | -0.18 | 0.001 | -0.066 | 0.22 | -0.13 | 0.023 | -0.16 | 0.004 | -0.18 | 0.002 |
| cg25737313 | -0.28 | 4.8E-07 | -0.22 | 9.9E-06 | -0.17 | 0.001 | -0.14 | 0.009 | -0.056 | 0.27 | -0.16 | 0.002 | -0.15 | 0.004 |
| cg08274633 | -0.42 | 1.5E-08 | -0.28 | 3.1E-05 | -0.3 | 2.1E-05 | -0.12 | 0.093 | -0.16 | 0.027 | -0.25 | 4.6E-04 | -0.25 | 3.4E-04 |
| cg01695954 | -0.40 | 5.7E-09 | -0.26 | 3.6E-05 | -0.34 | 1.9E-07 | -0.17 | 0.008 | -0.20 | 0.003 | -0.27 | 5.1E-05 | -0.059 | 0.36 |
| cg24704287 | -0.66 | 5.5E-08 | -0.37 | 8.8E-04 | -0.48 | 4.3E-05 | -0.31 | 0.009 | -0.28 | 0.017 | -0.42 | 2.2E-04 | -0.29 | 0.013 |
| cg20559385 | -0.61 | 8.8E-07 | -0.52 | 5.5E-06 | -0.25 | 0.038 | -0.36 | 0.002 | -0.25 | 0.031 | -0.34 | 0.004 | -0.25 | 0.032 |
| cg01766737 | -0.13 | 4.2E-07 | -0.10 | 1.8E-05 | -0.095 | 9.4E-05 | -0.039 | 0.11 | -0.069 | 0.004 | -0.069 | 0.005 | -0.044 | 0.064 |
| cg12581512 | -0.29 | 6.7E-07 | -0.23 | 2.3E-05 | -0.21 | 1.4E-04 | -0.071 | 0.20 | -0.13 | 0.021 | -0.12 | 0.036 | -0.18 | 0.001 |
| cg04803208 | 0.51 | 9.4E-07 | 0.32 | 8.1E-04 | 0.31 | 0.003 | 0.32 | 0.001 | 0.14 | 0.17 | 0.38 | 1.8E-04 | 0.21 | 0.034 |
| Beta is the regression coefficient representing the percent change in DNA methylation associated with a 1 standard deviation increase in the psychosocial doman.  Models: CpG sites ~ Stress Domain + Age + Sex + Educational Attainment + Interview Year + Genetic Ancestry PC 1-10 + White Blood Cell Proportions + Row Position on Chip + Column Position on Chip + Sample Place | | | | | | | | | | | | | | |

| Supplemental Table 6: Total variability of CpG sites identified in Model 1 (FDR q<0.10) explained by cumulative psychosocial stress | | |
| --- | --- | --- |
| **CpG Sites** | **Percent of Variance Explained by Cumulative Psychosocial Stress^a^** | |
|  | *Model 1* | *Model 2* |
| cg17565444 | 0.97% | 0.80% |
| cg25737313 | 0.72% | 0.61% |
| cg08274633 | 1.91% | 1.78% |
| cg01695954 | 1.19% | 1.00% |
| cg24704287 | 0.88% | 0.65% |
| cg20559385 | 1.72% | 0.90% |
| cg01766737 | 1.65% | 1.22% |
| cg12581512 | 0.62% | 0.49% |
| cg04803208 | 4.82% | 4.49% |
| Model 1: CpG Site ~ Psychosocial Stress Score + Age + Sex + Educational Attainment + Interview Year + Genetic Ancestry PC 1-10 + White Blood Cell Proportions + Row Position on Chip + Column Position on Chip + Sample Plate  Model 2: Model 1 + Marital Status + Employment + Has Children + Total Household Wealth  ^a^ Variance explained is the difference in R^2^ between a full model (includes cumulative psychosocial stress score) and a reduced model (excludes cumulative psychosocial stress score). | | |

| Supplemental Table 7: Mediation of health behaviors on the relationship between cumulative psychosocial stress and CpGs identified in Model 1 (FDR q<0.10) | | | | | | | | | | |  |
| --- | --- | --- | --- | --- | --- | --- | --- | --- | --- | --- | --- |
|  | cg17565444 | | cg25737313 | | cg08274633 | | cg01695954 | | cg24704287 | |  |
|  | Beta | % Mediated | Beta | % Mediated | Beta | % Mediated | Beta | % Mediated | Beta | % Mediated |  |
| Total Effect | **-0.29** | **100%** | **-0.25** | **100%** | **-0.41** | **100%** | **-0.36** | **100%** | **-0.55** | **100%** |  |
| Direct Effect | **-0.24** | **82.76%** | **-0.22** | **88.00%** | **-0.37** | **90.24%** | **-0.29** | **80.56%** | **-0.43** | **78.18%** |  |
| Indirect Effect | **-0.05** | **17.24%** | **-0.04** | **12.00%** | **-0.04** | **9.76%** | **-0.06** | **19.44%** | **-0.12** | **21.82%** |  |
| Via Former Smoker | -0.005 | 1.66% | -0.0009 | 0.38% | -0.002 | 0.60% | -0.0069 | 1.93% | -0.02 | 2.98% |  |
| Via Current Smoker | **-0.03** | **8.77%** | -0.01 | 4.44% | -0.008 | 1.84% | **-0.0246** | **6.89%** | -0.04 | 7.45% |  |
| Via Moderate Alcohol Use | -0.003 | 0.98% | -0.007 | 2.67% | -0.01 | 2.94% | 0.0026 | -0.72% | 0.001 | 0.21% |  |
| Via Heavy Alcohol Use | -0.0003 | 0.11% | -0.0008 | 0.33% | -0.001 | 0.24% | 0.0006 | -0.16% | 0.0003 | -0.05% |  |
| Via Physically Active | -0.07 | 2.28% | 0.002 | -0.81% | -0.005 | 1.16% | -0.0076 | 2.14% | 0.005 | 0.89% |  |
| Via BMI | -0.005 | 2.73% | **-0.02** | **6.67%** | **-0.02** | **3.96%** | **-0.0260** | **7.30%** | **-0.06** | **10.37%** |  |
|  | cg01766737 | | cg12581512 | | cg20559385 | | cg04803208 | |  |  |  |
|  | Beta | % Mediated | Beta | % Mediated | Beta | % Mediated | Beta | % Mediated |  |  |  |
| Total Effect | **-0.11** | **100%** | **-0.26** | **100%** | **-0.64** | **100%** | **0.49** | **100%** |  |  |  |
| Direct Effect | **-0.09** | **81.87%** | **-0.21** | **80.77%** | **-0.58** | **90.63%** | **0.40** | **81.63%** |  |  |  |
| Indirect Effect | **-0.02** | **18.13%** | **-0.05** | **19.23%** | **-0.06** | **9.38%** | **0.09** | **18.37%** |  |  |  |
| Via Former Smoker | -0.002 | 1.72% | -0.002 | 0.88% | 0.004 | -0.69% | 0.005 | 1.05% |  |  |  |
| Via Current Smoker | **-0.006** | **5.42%** | **-0.02** | **7.67%** | -0.02 | 2.56% | 0.008 | 1.65% |  |  |  |
| Via Moderate Alcohol Use | -0.003 | 2.45% | -0.002 | 0.93% | -0.01 | 1.87% | **0.02** | **3.90%** |  |  |  |
| Via Heavy Alcohol Use | -0.0002 | 0.22% | -0.0003 | 0.10% | 0.0007 | -0.11% | 0.00006 | 0.01% |  |  |  |
| Via Physically Active | 0.0007 | 0.62% | 0.002 | 0.67% | -0.01 | 1.69% | 0.01 | 3.00% |  |  |  |
| Via BMI | **-0.008** | **7.03%** | **-0.021** | **8.49%** | -0.02 | 3.44% | **0.04** | **8.38%** |  |  |  |
| Beta is the regression coefficient representing the percent change in DNA methylation associated with a 1 point increase in health behavior. The CpG sites have been pre-adjusted for bath effects( row position on chip, column position on chip, sample plate) and white blood cell proportions. | | | | | | | | | | | |
| Bolded values represent significant associations at p<0.05. | | | | |  |  |  |  |  |  |  |
| Models: CpG Residuals~ Psychosocial Stress Score + Age + Sex + Educational Attainment + Marital Status + Employment + Has Children + Total Household  Wealth + Smoking + Alcohol + Physical Activity + BMI + Interview Year + Genetic Ancestry PC 1-10 | | | | | | | | | | | |

| Supplemental Table 8: Mediation of health behaviors measured in 2014 on the relationship between cumulative psychosocial stress and CpGs identified in Model 1 (FDR q<0.10) (N=2614) | | | | | | | | | | |
| --- | --- | --- | --- | --- | --- | --- | --- | --- | --- | --- |
|  | cg17565444 | | cg25737313 | | cg08274633 | | cg01695954 | | cg24704287 | |
|  | Beta | % Mediated | Beta | % Mediated | Beta | % Mediated | Beta | % Mediated | Beta | % Mediated |
| Total Effect | **-0.29** | **100%** | **-0.26** | **100%** | **-0.42** | **100%** | **-0.36** | **100%** | **-0.55** | **100%** |
| Direct Effect | **-0.24** | **81.19%** | **-0.22** | **84.17%** | **-0.36** | **85.71%** | **-0.28** | **77.66%** | **-0.41** | **74.02%** |
| Indirect Effect | **-0.05** | **18.81%** | **-0.04** | **15.83%** | **-0.06** | **14.29%** | **-0.08** | **22.34%** | **-0.14** | **25.98%** |
| Via Former Smoker | -0.004 | 1.21% | -0.001 | 0.39% | -0.003 | 0.80% | -0.007 | 1.95% | -0.01 | 2.59% |
| Via Current Smoker | **-0.03** | **11.53%** | **-0.01** | **5.45%** | -0.008 | 2.03% | **0.03** | **8.26%** | **-0.05** | **8.98%** |
| Via Moderate Alcohol Use | -0.004 | -1.33% | -0.001 | 0.56% | -0.005 | 1.32% | 0.002 | -0.58% | 0.004 | -0.80% |
| Via Heavy Alcohol Use | -0.001 | 0.34% | -0.002 | 0.75% | -0.005 | 1.15% | 0.0008 | -0.21% | 0.004 | -0.65% |
| Via Physically Active | -0.009 | 3.23% | -0.004 | 1.65% | -0.01 | 3.36% | **-0.01** | **4.00%** | **-0.03** | **5.09%** |
| Via BMI | -0.01 | 3.83% | **-0.02** | **7.04%** | **-0.02** | **5.64%** | **-0.03** | **8.91%** | **-0.06** | **10.98%** |
|  | cg01766737 | | cg12581512 | | cg20559385 | | cg04803208 | |  |  |
|  | Beta | % Mediated | Beta | % Mediated | Beta | % Mediated | Beta | % Mediated |  |  |
| Total Effect | **-0.10** | **100%** | **-0.26** | **100%** | **-0.65** | **100%** | **0.49** | **100%** |  |  |
| Direct Effect | **-0.08** | **81.64%** | **-0.19** | **74.06%** | **-0.58** | **89.93%** | **0.39** | **80.01%** |  |  |
| Indirect Effect | **-0.02** | **18.36%** | **-0.07** | **25.94%** | **-0.07** | **10.07%** | **0.10** | **19.99%** |  |  |
| Via Former Smoker | -0.002 | 1.58% | -0.001 | 0.57% | 0.005 | -0.73% | 0.006 | 1.14% |  |  |
| Via Current Smoker | **-0.006** | **5.57%** | **-0.03** | **10.40%** | -0.02 | 3.36% | 0.008 | 1.67% |  |  |
| Via Moderate Alcohol Use | -0.0005 | 0.51% | -0.0008 | 0.32% | -0.004 | 0.65% | 0.006 | 1.34% |  |  |
| Via Heavy Alcohol Use | -0.0007 | 0.71% | -0.003 | 1.05% | 0.006 | -0.92% | 0.007 | 1.51% |  |  |
| Via Physically Active | -0.003 | 3.06% | **-0.01** | **4.71%** | **-0.03** | **3.79%** | 0.02 | 4.11% |  |  |
| Via BMI | **-0.007** | **6.93%** | **-0.02** | **8.89%** | -0.03 | 3.92% | **0.05** | **10.23%** |  |  |
| Beta is the regression coefficient representing the percent change in DNA methylation associated with a 1 point increase in health behavior. The CpG sites have been pre-adjusted for bath effects( row position on chip, column position on chip, sample plate) and white blood cell proportions. | | | | | | | | | | |
| Bolded values represent significant associations at p<0.05. | | | |  |  |  |  |  |  |  |
| Models: CpG Residuals~ Psychosocial Stress Score + Age + Sex + Educational Attainment + Marital Status + Employment + Has Children + Total Household  Wealth + Smoking + Alcohol + Physical Activity + BMI + Interview Year + Genetic Ancestry PC 1-10 | | | | | | | | | | |
